# Multimodal Imaging of Systemic Metastatic Myocardial and Vascular Calcification Associated with Renal Secondary Hyperparathyroidism in a Castrated Male Cat with End-Stage Chronic Kidney Disease: A Case Report

**DOI:** 10.3390/ani16081169

**Published:** 2026-04-10

**Authors:** Minsoo Chung, Jungmin Kwak, Suhyung Lee, Kidong Eom, Jaehwan Kim

**Affiliations:** 1 Department of Veterinary Medical Imaging, College of Veterinary Medicine, Konkuk University, Seoul 05029, Republic of Korea; ms3871@konkuk.ac.kr (M.C.); eomkd@konkuk.ac.kr (K.E.); 2 Human & Animal Medical Center, Yongin 16843, Republic of Korea; chocojae@naver.com; 3 Department of Veterinary Pathology, College of Veterinary Medicine, Konkuk University, Seoul 05029, Republic of Korea; suhyunglee@konkuk.ac.kr

**Keywords:** calcium–phosphorus product, cat, chronic kidney disease, computed tomography, echocardiography, metastatic calcification, uremic cardiomyopathy, vascular calcification

## Abstract

Myocardial calcification is an uncommon complication associated with end-stage chronic kidney disease (CKD) in feline patients. This report describes the clinical and multimodal imaging features of metastatic calcification in a 10-year-old castrated male mixed-breed cat. The patient presented with dyspnea and anorexia, and was diagnosed with IRIS Stage 4 CKD. Laboratory findings revealed severe hyperphosphatemia and an elevated calcium–phosphorus product (CPP) of 135 mg^2^/dL^2^, based on total calcium. This value significantly exceeds 70 mg^2^/dL^2^, a threshold associated with a high probability of inducing soft tissue mineralization. Echocardiography revealed extensive hyperechoic foci with posterior acoustic shadowing in the interventricular septum and left ventricular wall. Functional assessment demonstrated a restrictive diastolic filling pattern, suggesting increased myocardial stiffness and congestive heart failure. Computed tomography (CT) further visualized systemic involvement, showing diffuse, amorphous calcifications (400–900 HU) in the myocardium, multifocal aortic wall, and extracardiac tissues. Despite intensive treatment with diuretics and renal support, the patient was euthanized eight days later due to progressive renal failure. This case illustrates that the interaction between metastatic calcification and uremic cardiomyopathy (UC) can result in refractory heart failure, underscoring the value of combined echocardiography and CT in evaluating end-stage renal disease.

## Simple Summary

Myocardial calcification is an uncommon but life-threatening complication in cats with advanced kidney disease. This condition occurs when minerals such as calcium and phosphorus accumulate in the myocardial tissue due to impaired renal filtration, resulting in increased myocardial stiffness and subsequent diastolic and systolic dysfunction. This report describes a 10-year-old castrated male cat that developed congestive heart failure, accelerated by extensive mineral deposits in the myocardium and great vessels. Echocardiography and computed tomography were used to assess the extent of these deposits and their impact on cardiac function. Despite intensive medical treatment aimed at supporting both the renal and cardiac function, the pathological changes were irreversible, eventually leading to euthanasia. This study characterizes this uncommon complication, helping veterinarians to better understand and diagnose this condition in cats with end-stage renal failure.

## 1. Introduction

Myocardial calcification is an uncommon pathological finding in feline medicine [1,2]. It is generally classified into three forms—dystrophic, metastatic, and idiopathic—based on the underlying etiopathogenesis [2,3,4]. Dystrophic myocardial calcification occurs in locally damaged or necrotic cardiac tissues, often secondary to myocardial infarction, myocarditis (viral or fungal), sepsis, inflammatory processes (e.g., rheumatic heart disease), neoplasia (e.g., metastatic or primary cardiac tumors), or cardiac trauma [2,3,4]. In these cases, serum calcium levels typically remain within the normal range [2,3].

In contrast to dystrophic calcification, metastatic calcification results from systemic metabolic disturbances that disrupt calcium–phosphorus homeostasis, most notably in end-stage chronic kidney disease (CKD) [2,3,4]. Metastatic calcification can affect previously healthy myocardial tissue and is characterized by a more diffuse and amorphous distribution [3]. In CKD, this process is primarily driven by a significant decline in the glomerular filtration rate, which leads to hyperphosphatemia as the kidneys fail to adequately excrete excess phosphate [5]. To regulate elevated phosphate levels, the body increases fibroblast growth factor-23 (FGF-23) secretion, which subsequently reduces the concentration of active vitamin D [5]. This vitamin D deficiency decreases intestinal calcium absorption, triggering a compensatory surge in parathyroid hormone (PTH) known as renal secondary hyperparathyroidism [5]. Excessive PTH secretion strongly stimulates bone resorption, releasing large amounts of calcium and phosphorus from the skeletal system into the bloodstream [5,6]. When the serum calcium–phosphorus product exceeds the capacity of the skeletal system to accommodate the surplus ions, these minerals bind and precipitate as hydroxyapatite crystals [5]. While calcification in non-visceral sites such as arteries consists of hydroxyapatite, visceral calcification in the heart and lungs often exhibits an amorphous structure composed of calcium, magnesium, and phosphorus [7]. Furthermore, elevated PTH can directly induce calcium influx into cardiomyocytes by activating G-protein signaling, a mechanism that can independently trigger metastatic myocardial calcification even when serum calcium levels are normal [3,8]. Beyond CKD, severe electrolyte imbalances caused by primary hyperparathyroidism, vitamin D toxicosis, osteolytic malignancies (e.g., osteosarcoma), and paraneoplastic syndromes can also induce metastatic calcification through similar mechanisms [3,4].

In feline patients, CKD can significantly impact cardiac health through a pathological interaction known as cardiovascular–renal disorders (CvRDs) [9]. Specifically, when CKD leads to secondary cardiac injury, it is classified as CvRD_k_ [9]. The mechanisms driving this progression are multifactorial. In uremic cats, anorexia, hypodipsia, and vomiting often cause volume depletion, resulting in reduced cardiac output [9]. Conversely, kidney injury can also lead to systemic volume overload, contributing to congestion, especially in animals with coexisting conditions such as diastolic dysfunction or severe anemia [9]. Furthermore, systemic hypertension, a common consequence of CKD, frequently results in myocardial hypertrophy and dysfunction [9]. Finally, azotemia itself may exert direct adverse effects on cardiac myocytes [9]. While these hemodynamic and metabolic factors are known to influence the heart, the specific role of extensive myocardial mineralization in promoting restrictive functional changes remains incompletely understood in veterinary literature.

Myocardial calcification is a recognized complication of chronic metabolic derangements; however, its clinical presentation and diagnostic imaging features in feline patients remain insufficiently documented. The objective of this case report is to describe the clinical, laboratory, and multimodal imaging findings—including radiography, echocardiography, and computed tomography (CT)—of extensive myocardial calcification in a cat with end-stage CKD. By correlating functional cardiac impairment with structural mineralization, this report aims to provide clinical insights into the complexities of managing the cardiorenal dilemma in feline medicine.

## 2. Case Description

### 2.1. Signalment, History, and Clinical Findings

A 10-year-old castrated male mixed-breed cat was presented with a 3-day history of lethargy and anorexia. For the preceding six months, the patient had been managed for International Renal Interest Society (IRIS) stage 3 CKD. The management protocol included twice-daily subcutaneous fluid therapy with 0.45% saline (90 mL/cat per dose; total 180 mL/day) to maintain adequate hydration while minimizing the cumulative sodium load. Each dose was administered over approximately 5 to 10 min to avoid potential complications associated with rapid volume overload. During this period, the patient’s systolic blood pressure remained stable between 150 and 159 mmHg; therefore, ACE inhibitors or other vasodilators had not been prescribed, as the hypertension was considered well-managed within the borderline range.

On physical examination, the patient was obtunded, with a body condition score of 3/9 and concurrent generalized muscle wasting. Although thoracic auscultation revealed no specific abnormalities and the heart rate was within normal limits, the patient exhibited tachypnea, with a respiratory rate of 48 breaths per minute. Additionally, the patient was hypertensive, with a systolic blood pressure of 170 mmHg.

At presentation, laboratory evaluations revealed severe azotemia, hyperphosphatemia, and mild to moderate hyperkalemia, accompanied by metabolic acidosis. Although the total calcium concentration remained within the normal range, ionized hypocalcemia was present. These metabolic disturbances were further complicated by significant hypoproteinemia and hypoalbuminemia. Additionally, the combination of markedly elevated serum PTH and low 25-hydroxyvitamin D concentrations confirmed a diagnosis of renal secondary hyperparathyroidism. Detailed laboratory values and their respective reference ranges are summarized in Table 1. The calculated calcium–phosphorus product (CPP) was 135 mg^2^/dL^2^ based on total calcium, while the ionized calcium (iCa)-based CPP was 63 mg^2^/dL^2^; both values significantly exceeded their respective clinical thresholds, indicating a severe predisposition to metastatic soft tissue calcification. A urinalysis was not performed during the acute presentation, as previous results from six months prior had already demonstrated persistent isosthenuria (USG 1.008 [1.035–1.060]) and significant proteinuria (3+), which were considered sufficient evidence of irreversible renal dysfunction at that time.

### 2.2. Imaging, Diagnosis and Outcome

**Figure 1:**
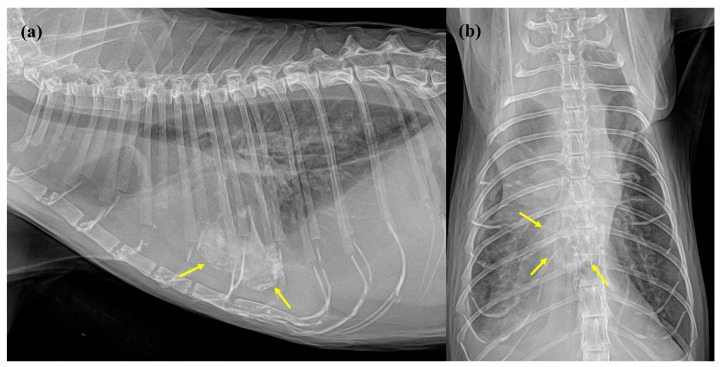
Initial thoracic radiographs at presentation: (**a**) Right lateral view. Severe pleural effusion is evidenced by increased soft tissue opacity in the ventral thorax, accompanied by silhouetting of the cardiac borders. A prominent, heterogeneously mineralized, globoid-shaped mass (yellow arrows) is identified in the ventral region of the obscured cardiac silhouette, extending from the 5th to the 7th intercostal spaces. (**b**) Ventrodorsal view. The mineralized opacity (yellow arrows) is located in the mid-sagittal plane at the level of the 9th to 11th thoracic vertebrae, superimposed over the cardiac apex. A concurrent unstructured interstitial pattern is visible in the right caudal lung lobe.

Thoracic radiographs (HF-525Plus VET; Eco-ray, Seoul, Republic of Korea; 55 kVp, 5 mAs) were obtained. In the right lateral view, increased soft tissue opacity was observed in the ventral thorax with subsequent effacement of the cardiac silhouette, consistent with pleural effusion (Figure 1a). Multifocal subcutaneous emphysema was also noted in the dorsal subcutaneous region. Notably, a markedly mineralized, heterogeneous, globoid-shaped mass was identified in the ventral region of the obscured cardiac silhouette, localized at the level of the 5th to 7th intercostal spaces. In the ventrodorsal view, this opacity was positioned in the mid-sagittal plane at the level of the 9th to 11th thoracic vertebrae, superimposed over the cardiac apex (Figure 1b). Additionally, an unstructured interstitial pattern was identified in the right caudal lung lobe. Differential diagnoses for the pulmonary interstitial change included cardiogenic pulmonary edema, uremic pneumonitis, or pulmonary edema of renal origin. The subcutaneous emphysema was considered an iatrogenic change secondary to chronic subcutaneous fluid therapy. Regarding the mineralized mass, metabolic, neoplastic, inflammatory, or infectious etiologies were considered, manifesting as either dystrophic or metastatic calcification. Therapeutic thoracocentesis was performed to alleviate respiratory distress caused by pleural effusion; subsequent fluid analysis characterized the effusion as a pure transudate.

**Figure 2:**
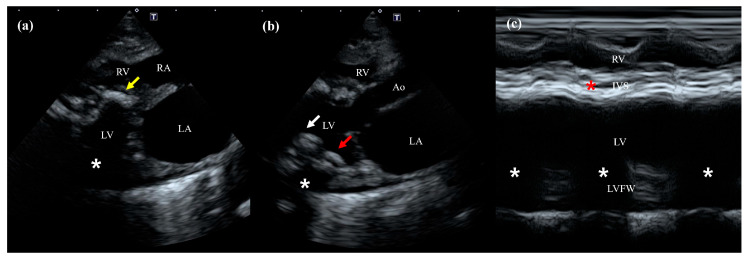
Two-dimensional and M-mode echocardiographic images from a right parasternal long-axis view. (**a**) Systolic frame showing extensive, irregular hyperechoic foci (yellow arrow) within the interventricular septum (IVS). (**b**) Diastolic frame revealing similar hyperechoic foci localized in the left ventricular free wall (LVFW) (red arrow) and papillary muscle (white arrow). In both (**a**) and (**b**), prominent posterior acoustic shadowing (asterisks) is observed secondary to dense myocardial mineralization, along with marked left atrial enlargement. (**c**) M-mode image demonstrating restricted motion (akinesis) of the calcified IVS (red asterisk), with a marked reduction in both systolic and diastolic excursions. Note that the endocardial excursion of the LVFW is partially obscured by significant acoustic shadowing (white asterisks) originating from the septal and ventricular wall mineralization. Abbreviations: LA, left atrium; LV, left ventricle; RA, right atrium; RV, right ventricle; Ao, aorta; IVS, interventricular septum; LVFW, left ventricular free wall.

Two-dimensional transthoracic echocardiography was performed using a 5-MHz phased-array transducer (Aplio i800; Canon Medical Systems, Zoetermeer, The Netherlands). Echocardiography revealed multifocal, irregular, hyperechoic foci consistent with mineralization within multiple cardiac structures, including the interventricular septum (IVS), left ventricular (LV) free wall, and papillary muscles (Figure 2a,b). Notably, mineralization was most severe within the IVS. These mineralized areas caused prominent posterior acoustic shadowing, which hindered precise cardiac evaluation via M-mode echocardiography in most views (Figure 2c). Consequently, quantitative systolic function indices such as fractional shortening and ejection fraction could not be reliably measured. Therefore, the assessment of systolic function was limited to a subjective evaluation of real-time cine loops, which revealed localized akinesis of the affected wall and decreased left ventricular contractility compared to the normal systolic indices observed six months earlier. The thickness of the interventricular septum (6.8 mm) and the left ventricular free wall (6.3 mm) remained stable compared to the previous examination. However, in contrast to the previously normal left atrial (LA) size (10.5 mm), the current evaluation revealed significant enlargement, with a maximum diastolic LA diameter of 17.8 mm (reference range: <16 mm [10]) and a left atrial-to-aortic diameter ratio of 1.88 (reference range: <1.5 [11]). The heart rate was 180 bpm, which was considered within the normal range. Assessment via spectral and tissue Doppler imaging revealed a mitral E/A ratio of 2.8 (reference range: <2 [11,12]), an isovolumic relaxation time (IVRT) of 30 ms (reference range: >60 ms [13]), and an E/E’ ratio (LV free wall) of 10.0 (reference range: <10 [14]); moreover, no E/A fusion was observed. Consequently, although volume overload due to fluid therapy could not be entirely excluded, the overall findings were suggestive of a restrictive diastolic pattern.

Therapeutic intervention was implemented using a staged approach, closely guided by the patient’s evolving clinical status. Initially, to manage severe uremia and hyperphosphatemia, fluid therapy (Hartmandex solution supplemented with vitamin B and glutathione) was initiated at 10 mL/h. This was combined with aluminum hydroxide (15 mg/kg, PO, q12h) as a phosphate binder and an oral spherical carbon adsorbent (Renamezin^®^; 100 mg/kg, PO, q12h).

Following echocardiographic findings of myocardial mineralization, decreased contractility, and congestive heart failure (CHF), the treatment strategy was immediately adjusted to address the therapeutic challenges of concurrent heart and kidney failure. To alleviate pulmonary edema and reduce cardiac preload, intravenous furosemide (1 mg/kg, q12h) was administered, and the fluid rate was strategically reduced from 10 mL/h to 4.5 mL/h (approximately 50% of the maintenance rate) to prevent further volume overload. Simultaneously, pimobendan (0.25 mg/kg, PO, q12h) was introduced to improve myocardial contractility [15]. Despite the presence of hyperkalemia, spironolactone (2 mg/kg, PO, q12h) was administered to mitigate cardiac, renal, and vascular remodeling [9,16]. Given that spironolactone is a potassium-sparing diuretic, serum potassium levels were closely monitored to ensure patient safety and prevent further electrolyte imbalance. Despite the initiation of spironolactone, the serum potassium concentration progressively decreased from an initial 5.61 mmol/L to 4.93 mmol/L at the final measurement before death, likely due to the concurrent potassium-wasting effect of aggressive furosemide therapy [17]. Due to persistently uncontrolled hyperphosphatemia, the dosage of aluminum hydroxide was increased to 30 mg/kg PO every 12 h. Furthermore, sevelamer, a non-calcium-based phosphate binder, was added to the regimen at 20 mg/kg PO twice daily to achieve more synergistic phosphorus control and mitigate further mineral deposition.

Regarding the management of renal anemia, the patient had been receiving long-term treatment with darbepoetin (0.5 µg/kg, SC, biweekly) and iron dextran (10 mg/kg, IM, biweekly) for the previous six months, with a single dose administered during the hospitalization period.

Despite intensive inpatient therapeutic efforts, no significant clinical improvement was observed, and the patient’s renal function continued to deteriorate progressively. Consequently, due to the grave prognosis and lack of response to treatment, the owner elected euthanasia eight days after the initial identification of myocardial mineralization.

Immediately post-mortem, with the owner’s informed consent, a computed tomography (CT) scan was performed to further elucidate the underlying etiopathogenesis of the mineralization. The scan (Brilliance CT 16-slice; Philips, Amsterdam, The Netherlands) was conducted with the patient in sternal recumbency using the following parameters: 120 kVp, 150 mAs, 512 × 512 matrix, 1 mm slice thickness, 0.75 s rotation time, and a collimation beam pitch of 0.9. Pre-contrast images of the thoracic and cranial abdominal regions were obtained and reconstructed using a soft tissue algorithm in transverse, dorsal, and sagittal planes. Images were reviewed using a soft tissue window (WW 400, WL 60), a bone window (WW 1500, WL 300), and a lung window (WW 1600, WL −600). Three-dimensional volume rendering was performed using post-processing software (RadiAnt DICOM Viewer, version 2020.2.3; Medixant, Poznań, Poland).

To quantify mineral density, Hounsfield units (HUs) were measured using circular regions of interest (ROIs; 1–10 mm^2^). Due to the irregular and heterogeneous distribution of mineralization, ROIs were selectively placed at the center of the most hyperattenuating foci to minimize partial volume effects from adjacent soft tissues. To ensure repeatability and methodological transparency, each site was measured three times in slightly different planes, and the final recorded mean HU value was calculated from these repetitions. All ROI placements and measurements were performed by a primary observer (a veterinary radiologist with five years of experience) and subsequently verified by a senior observer (a veterinary radiologist with 14 years of experience) to ensure consistency and minimize inter-observer variability.

**Figure 3:**
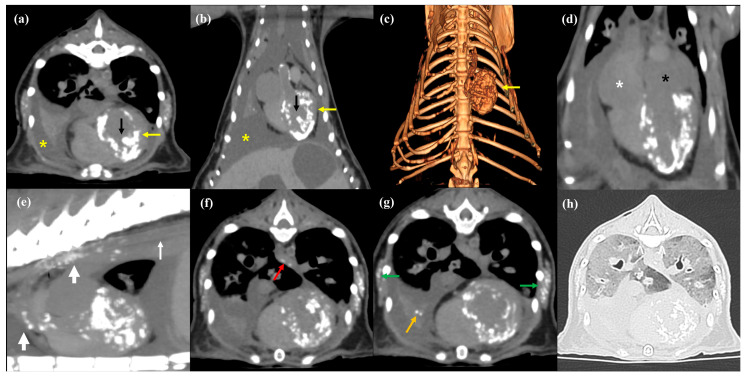
Post-mortem CT images. (**a**–**c**) Axial, coronal, and volume-rendered images, respectively, demonstrating diffuse, amorphous, and heterogeneous hyperattenuating calcifications within the left ventricle (yellow arrows) and papillary muscles (black arrows), accompanied by generalized pleural effusion (yellow asterisks). (**d**) Coronal image showing marked biatrial enlargement (white asterisk, right atrium; black asterisk, left atrium). (**e**) Maximum intensity projection sagittal image revealing extensive calcification along the ventral and dorsal walls of the thoracic aorta (thin white arrow), with localized areas of prominent mineral deposition within the ascending and descending aorta (thick white arrows). (**f**) Small, focal calcified lesions within the ventral wall of the intrathoracic esophagus (red arrow). (**g**) Calcification within the completely collapsed right middle lung lobe (orange arrow) and generalized calcification of the bilateral serratus muscles (green arrows). (**h**) Lung window image demonstrating diffuse ground-glass opacities (GGOs) and generalized bronchial wall thickening across the entire lung fields.

CT revealed extensive multisystemic calcification. Cardiovascular lesions involved the left ventricle, papillary muscles, and multiple segments of the aortic wall, where multifocal mineralized foci were identified (Figure 3a–e). These lesions appeared as diffuse, amorphous, and heterogeneous hyperattenuating foci with bone-equivalent densities (Hounsfield units [HUs]: 400–900) (Figure 3). Secondary to these chronic changes, severe biatrial enlargement was also observed, consistent with the pressure and volume overload often associated with restrictive cardiac pathology.

Furthermore, multiple small, calcified foci were identified in extra-cardiac tissues, including the esophageal wall and various skeletal muscles of the thorax and spine (e.g., bilateral serratus, infraspinatus, subscapularis, and the epaxial and hypaxial musculature) (Figure 3g). The visualized skeletal structures, including the vertebrae and ribs, showed no significant abnormalities in bone mineral density or structural integrity, with no evidence of renal osteodystrophy secondary to CKD. In the cranial abdomen, three large, well-defined, hypoattenuating cysts (HU: 5–10) were identified within the hepatic parenchyma. Given the previous ultrasonographic identification of multiple bilateral renal cysts, these findings are highly suggestive of polycystic kidney disease (PKD), which likely contributed to the progression of the patient’s CKD. Additionally, large-volume pleural effusion (HU: 5–12) and a small amount of ascites (HU: 5–8) were present. Although histopathological confirmation was not performed, a presumptive diagnosis of systemic metastatic calcification secondary to end-stage CKD was considered based on these comprehensive imaging and laboratory findings.

Regarding the lung parenchyma, diffuse ground-glass opacities (GGOs) were observed throughout the lobes, accompanied by complete consolidation of the right middle lung lobe and generalized bronchial wall thickening. While the GGOs may partially represent post-mortem atelectasis or hypostatic congestion, these findings are consistent with pre-existing pulmonary edema and uremic pneumonitis identified on antemortem radiographs. Potential differential diagnoses for the bronchial wall thickening include chronic primary bronchitis, metastatic calcification of the bronchial tree, or chronic peribronchial edema. Further gross necropsy and histopathological evaluation were not performed per the owner’s request.

## 3. Discussion

In this present case, the patient’s long-standing history of CKD, hyperphosphatemia, and a markedly elevated calcium–phosphorus product (CPP) of 135 mg^2^/dL^2^ provide a strong clinical basis for a presumptive diagnosis of metastatic calcification. Given the absence of histopathological confirmation, this diagnosis is primarily supported by these metabolic disturbances combined with characteristic multimodal imaging findings. The CPP serves as a critical clinical indicator for predicting the risk of cardiovascular disease and metastatic calcification; maintaining a CPP below 55 mg^2^/dL^2^ is recommended [18], while a value exceeding 70 mg^2^/dL^2^ is associated with a sharply increased incidence of extensive myocardial calcification [2,18]. In this case, despite a normal total serum calcium concentration, ionized calcium (iCa)—the biologically active form—was additionally measured to provide a more physiologically relevant assessment of the mineralizing risk [18]. Since total calcium is commonly bound to albumin, iCa-based CPP provides a more accurate reflection of the ionic environment predisposing to crystal precipitation [18]. For iCa-based CPP, the high-risk threshold is adjusted to above 2.8 mmol^2^/L^2^ (approximately 45 mg^2^/dL^2^) [18]. In this patient, the iCa-based CPP was calculated at 5.08 mmol^2^/L^2^ (63 mg^2^/dL^2^), based on an iCa of 1.05 mmol/L and a phosphorus level of 15 mg/dL. The finding that the iCa-based CPP significantly exceeded the high-risk limit by approximately 1.8-fold—even in the presence of ionized hypocalcemia—indicates that extreme hyperphosphatemia was the primary driver increasing the risk of metastatic calcification. This metabolic profile provided a crucial clinical rationale for aggressive phosphate-lowering therapy, establishing phosphorus management as the critical intervention to mitigate further mineral deposition. Notably, once metastatic calcification is established due to a high CPP, the lesions exhibit an irreversible nature; subsequent normalization of serum CPP levels through treatment does not necessarily result in regression of existing calcific deposits [2,19]. Therefore, in managing patients with renal secondary hyperparathyroidism secondary to CKD, proactive monitoring of iCa-based CPP is clinically important to minimize the risk of metastatic calcification and to intervene before irreversible structural damage occurs.

While initial radiographs identified myocardial calcification, they were insufficient for precise anatomical localization or functional assessment. In this patient, echocardiography served as a crucial tool for evaluating the structural and hemodynamic consequences of calcification. The lesions appeared as distinct hyperechoic foci involving the interventricular septum, left ventricular free wall, and papillary muscles, accompanied by characteristic posterior acoustic shadowing. Functionally, the restrictive diastolic filling pattern and localized akinesis of the mineralized myocardial segments suggest that calcification significantly increased myocardial stiffness. This severe restrictive diastolic physiology was considered to result from the synergistic effect of extensive myocardial calcification and underlying myocardial fibrosis. Specifically, the myocardial fibrosis was attributed to uremic cardiomyopathy (UC), which has been shown in previous rat models to directly induce increased ventricular stiffness and fibrosis [20]. However, without a biopsy, it is difficult to determine the relative contributions of myocardial mineralization versus underlying fibrosis, and the latter may have played an even more predominant role in the restrictive filling pattern. Additionally, the possibility of CvRD_H_, where a primary cardiomyopathy might have coexisted with or contributed to renal progression, cannot be entirely excluded [9]. Ultimately, while extensive mineralization likely acted as a major factor, the impaired ventricular compliance should be considered a complex outcome of multiple concurrent pathologies, including uremic cardiomyopathy independent of calcification [21,22]. Although the patient did not exhibit the global systolic collapse (e.g., EF < 20–40%) reported in some severe cases [6,23], the presence of regional wall motion abnormalities indicates impaired systolic coordination. In this patient, however, this restrictive physiology appeared to be the primary driver of clinical decline, leading to increased filling pressures and subsequent life-threatening pleural effusion.

In the diagnostic clinical workflow for this patient, echocardiography played a pivotal role by identifying findings that were not apparent on thoracic radiographs. Specifically, it enabled the non-invasive detection of left atrial enlargement, diastolic dysfunction, and reduced systolic contractility. These functional assessments provided the clinical rationale for immediate therapeutic adjustments, such as reducing the fluid infusion rate to prevent volume overload and initiating a targeted regimen of furosemide, pimobendan, and spironolactone. However, echocardiography has inherent limitations in detecting calcified lesions. Compared to CT, its sensitivity for identifying mineralization is lower, and significant acoustic shadowing can restrict visualization of certain cardiac planes [3,23]. Therefore, while echocardiography remains the primary modality for assessing the functional consequences of cardiac calcification, it should ideally be complemented by CT to achieve a more definitive and localized anatomical diagnosis [23].

CT served as a critical complementary modality, providing a comprehensive “virtual necropsy” of the systemic extent of calcification. In human medicine, cardiac CT is the gold standard for detecting myocardial calcification due to its high spatial resolution and ability to quantify mineral density [2,23]. In contrast to the extensive research available in human cardiology, similar clinical studies in veterinary medicine remain limited; therefore, we extrapolated established human medical data to interpret the specific myocardial calcification patterns observed in this patient. The pattern and location of myocardial calcification on CT provide crucial insights into its underlying etiology [3]. Dystrophic calcification, typically resulting from local tissue injury, often appears as focal and linear patterns [3]. For instance, ischemic heart disease or myocardial infarction usually presents as thin curvilinear calcifications along the infarcted wall or isolated to the papillary muscles [3]. In contrast, infectious or inflammatory etiologies, such as sepsis or myocarditis, may result in circumferential linear or diffuse globular patterns that can potentially regress over time following successful treatment [3,6]. Furthermore, rheumatic heart disease typically localizes to the mitral annulus or basilar left ventricle in dense ring-like clumps, while pericarditis-related calcification presents as chunky linear deposits primarily involving the right heart and atrioventricular groove sparing the apex [3]. In this case, CT revealed diffuse, amorphous, and heterogeneous hyperattenuating foci (HU: 400–900) involving the left ventricle, papillary muscles, and multiple segments of the aorta. These morphological features—diffuse and amorphous rather than focal or thin-linear—are hallmarks of metastatic calcification [3]. Moreover, CT’s ability to detect extra-cardiac calcification in the esophageal wall, lungs, and skeletal muscles further confirmed the systemic nature of the metabolic disturbance, providing a level of detail unattainable with radiography or echocardiography. Unlike inflammatory forms, the extensive and systemic involvement observed here, driven by irreversible end-stage CKD, underscores the grave prognosis of metastatic calcification in feline patients. It should be noted that these CT-based patterns of myocardial calcification have not yet been extensively validated in veterinary patients. According to previous literature on cardiac and aortic calcification in dogs, dystrophic calcification—primarily resulting from age-related degenerative changes—manifests radiographically as either elongated lesions extending along the ascending aorta or short, curved lesions localized near the aortic valve [1]. These lesions often exhibit an irregular texture with small, plaque-like structures accumulating linearly, creating an appearance reminiscent of stacked coins [1]. On CT, this condition is characterized by distinct calcified bands encircling the ascending aorta and the proximal segment of the descending aorta [1]. However, in this case, we observed that the calcification patterns in this feline patient were consistent with those described as metastatic calcification in human literature. Nonetheless, due to the lack of histopathological confirmation and the inherent limitations of a single case report, it cannot be definitively concluded that all feline metastatic calcification will exhibit identical patterns. Therefore, further research is necessary to establish specific CT imaging patterns and diagnostic criteria for the various forms of myocardial mineralization in veterinary patients.

The management of this case presented a significant clinical dilemma. To address the uremia associated with end-stage CKD, fluid therapy was indicated; however, the presence of restrictive heart failure and pleural effusion necessitated the use of diuretics. Although furosemide carried a risk of exacerbating azotemia, it was employed as an essential rescue therapy to stabilize respiratory compromise. We aimed to maintain a delicate balance through conservative fluid rates and judicious diuretic use. Regarding renal anemia, proactive administration of darbepoetin and iron dextran was initiated based on the patient’s chronic history, aiming to provide long-term erythropoietic support despite the terminal nature of the crisis. Despite escalation of aluminum hydroxide and the addition of sevelamer to the therapeutic regimen, the patient’s serum phosphorus levels remained persistently elevated until death. In human medicine, several aggressive management strategies are established to mitigate the progression of metastatic calcification. First, pharmacological interventions emphasize the use of non-calcium-based phosphate binders, such as sevelamer or lanthanum carbonate, over calcium-containing agents to avoid increasing the total calcium burden [24]. The use of calcimimetics like cinacalcet is also preferred, as they effectively suppress PTH while simultaneously lowering or stabilizing serum calcium and phosphorus levels [24]. Regarding renal support, standard hemodialysis often has limitations in phosphorus clearance; therefore, more intensive protocols, including daily short-term or nocturnal dialysis, are employed to more effectively stabilize the calcium–phosphorus product [5]. While the therapeutic or preventive efficacy of these interventions has not yet been extensively established in veterinary patients with metastatic calcification, it can be inferred that—consistent with human medicine—early, multimodal phosphorus control and intensive metabolic stabilization are critical. These strategies may be essential to mitigate the progression of myocardial mineralization and could potentially improve clinical outcomes in feline patients facing similar cardiorenal dilemmas. It is reasonable to suggest that such intensive metabolic stabilization might play a pivotal role in managing this complex condition, although further clinical validation is needed.

In this case, the decision to initiate spironolactone—despite an initial potassium level of 5.61 mmol/L (reference range: 3.3–4.5 mmol/L)—was based on a multi-targeted therapeutic strategy aimed at achieving simultaneous diuresis, blood pressure control, and attenuation of RAAS-associated cardiac and vascular remodeling [16]. This approach was supported by the SEISICAT study, which demonstrated that the concurrent use of furosemide and spironolactone in cats with CHF does not significantly alter serum potassium concentrations compared to furosemide alone [16]. We anticipated that the potassium-wasting effect of aggressive furosemide therapy would counterbalance the potassium-sparing effect of spironolactone [16,17]. This hypothesis was validated by the patient’s subsequent electrolyte profile, where potassium levels progressively decreased to 4.93 mmol/L at the final measurement. Nevertheless, we acknowledge that administering a potassium-sparing diuretic in the presence of pre-existing hyperkalemia remains a clinically controversial and high-risk decision. While the anticipated cardiovascular benefits outweighed the risks under close monitoring in this specific case, this therapeutic dilemma underscores the need for extreme caution. Future studies and clinical guidelines should further address the safety of mineralocorticoid receptor antagonists in feline patients with concurrent severe renal and cardiac dysfunction to prevent potentially life-threatening electrolyte imbalances.

The prognosis of myocardial calcification varies by species. In dogs, it is often a benign, incidental, age-related change [1]. In cats, however, the clinical outlook appears far more severe, although this observation is based on a single published case report [2]. In that case, a 10-year-old cat remained stable for eight months following initial treatment but was ultimately euthanized due to progressive renal failure. Notably, although metastatic myocardial calcification driven by long-standing hyperphosphatemia induced congestive heart failure, the primary cause of death was the gradual and irreversible loss of renal function [2]. Unlike dystrophic calcification, which can sometimes provide mechanical stability post-infarction or regress after sepsis [6,25], metastatic calcification caused by irreversible end-stage CKD represents a terminal and refractory condition [3,26]. Consequently, despite potential temporary stabilization, the long-term prognosis is considered poor due to the inherently progressive and irreversible nature of the underlying systemic disturbance. Given that published cases describing the anatomical and functional consequences of this condition remain exceptionally uncommon in veterinary medicine, further research is warranted to establish a definitive prognostic profile for affected feline patients.

The pulmonary patterns observed in this case required a careful differential diagnosis among cardiogenic pulmonary edema, uremic pneumonitis, and pulmonary edema of renal origin. Classically, these conditions are characterized by a ‘batwing’ or ‘butterfly’ pattern—symmetrical opacities localized in the perihilar (central) regions while sparing the peripheral and basal lung fields [27]. However, in the present case, the radiographic findings included cardiomegaly, left atrial enlargement (LAE), and an asymmetrical interstitial pattern observed in the peripheral regions of the caudal lung lobes. These features align more closely with the typical feline CPE presentation—which frequently manifests as a multifocal distribution in the caudoventral and cranioventral fields; therefore, CPE was prioritized over uremic pneumonitis or renal-associated pulmonary edema [28]. However, interpreting these signs in the context of azotemia remains challenging. Unlike the classic central distribution seen in humans, research on azotemic dogs suggests that an alveolar pattern is the only pulmonary sign significantly associated with azotemia, and the characteristic ‘batwing’ distribution is notably absent [29]. Instead, azotemic lung lesions in small animals tend to be focal, often involving a single lung lobe rather than following a symmetrical perihilar pattern [29]. This clinical overlap suggests that, in feline patients with concurrent cardiorenal disease, radiographic patterns alone may be insufficient to definitively distinguish between uremic and cardiogenic origins. Consequently, further large-scale studies are warranted to establish specific thoracic radiographic patterns in cats with azotemia. Such research would be invaluable in developing more precise diagnostic criteria to differentiate uremic pneumonitis from cardiogenic pulmonary edema.

Regarding the etiology of the pleural effusion, uremic pleuropericarditis—a condition well-documented in human medicine—was considered a potential differential diagnosis given the patient’s severe uremic state. Uremic pleuropericarditis typically involves sterile, fibrinous inflammation caused by uremic toxins, resulting in an exudative and often serosanguineous effusion [27]. In the present case, however, the pleural fluid was characterized as a pure transudate. Therefore, the effusion was more likely a secondary hemodynamic consequence of congestive heart failure and hypoalbuminemia rather than a direct inflammatory response to uremia. To the authors’ knowledge, detailed reports or studies on the precise mechanisms and pathology of uremic pleuropericarditis in veterinary medicine remain scarce. Further research is warranted to elucidate whether similar uremia-induced inflammatory processes occur in veterinary patients.

In this present case, CT evaluation of the skull was not possible because the scan range was limited to the thorax and upper abdomen. However, no definitive signs of renal osteodystrophy (ROD) were observed on radiographic skull assessment or physical examination, and no cortical bone alterations were identified on the included CT images. In CKD, ROD typically manifests as fibrous osteodystrophy, in which elevated parathyroid hormone (PTH) levels stimulate osteoclasts to resorb bone, releasing calcium and phosphorus into the bloodstream [30,31]. This disrupts the balance between bone resorption and formation, leading to demineralization and replacement of bone with fibrous connective tissue [30,32]. Previous veterinary literature describes characteristic imaging features of ROD, such as widespread osteopenia of the skull and mandible, cortical thinning, and loss of alveolar bone, resulting in a ‘floating teeth’ appearance or the ‘rubber jaw’ phenomenon [31,32,33]. Although the exact prevalence of ROD in feline patients is not well documented, it is noted that while dogs frequently develop renal osteodystrophic disease, such manifestations are rarely reported in cats [34]. Several factors may explain the absence of overt ROD in this patient. First, ROD is most characteristic in young, growing animals with high bone turnover, where the immature skeleton is highly sensitive to the adverse effects of PTH [31,32,35]. As an older patient, this cat’s structurally mature skeleton may have been less responsive to elevated PTH levels. Second, species-specific factors in cats may result in a lower propensity for gross bone remodeling compared to dogs [30,33]. However, since a bone biopsy was not performed, the presence of subclinical or histopathological osteodystrophy cannot be entirely ruled out.

Several limitations exist in this study. The most significant limitation of this case report is the absence of histopathological confirmation through necropsy or biopsy. Although the markedly elevated calcium–phosphorus product and multimodal imaging findings strongly suggest metastatic calcification secondary to end-stage CKD, the lack of histological evaluation means that definitive differentiation from focal dystrophic calcification or other concurrent myocardial pathological processes (e.g., inflammation, neoplasia) cannot be entirely excluded. Specifically, dystrophic calcification might have coexisted in areas of chronic myocardial strain or underlying fibrosis. Therefore, although the systemic distribution of minerals supports a metabolic origin, the diagnosis of metastatic calcification in this report remains presumptive, based on clinical and imaging evidence rather than pathological confirmation. Additionally, the lack of electrocardiographic (ECG) evaluation is a notable limitation. Given that extensive myocardial mineralization and hyperkalemia—particularly within the IVS—can interrupt the cardiac conduction system and predispose patients to arrhythmias, ECG findings would have provided valuable clinically relevant information [36,37]. In this case, we prioritized identifying mineralization via advanced imaging, which consequently led to a secondary focus on electrophysiological assessment. However, future cases should incorporate ECG to provide a more comprehensive evaluation of functional consequences, specifically focusing on the correlation between structural myocardial calcification and potential electrical instability.

Furthermore, a notable limitation of this case is the absence of concurrent urinalysis during the acute crisis. Relying on six-month-old data fails to reflect the patient’s exact renal status at the time of myocardial calcification development. Specifically, the lack of current urinary data prevents assessment of the renal fractional excretion of minerals, as well as evaluation of current proteinuria levels, urine specific gravity, and the presence of urinary tract infections or inflammation. Additionally, the inability to identify crystalluria or casts through sediment analysis limits a more comprehensive understanding of ongoing mineral metabolism. These factors should be taken into account when interpreting the patient’s systemic mineralization and cardio-renal interactions.

Another limitation is the absence of cardiac biomarker measurements, such as N-terminal pro-B-type natriuretic peptide (NT-proBNP) and cardiac troponin I (cTnI), during the acute crisis. Although echocardiographic and CT findings provided evidence of structural and functional impairment, the lack of these biomarkers prevents a more objective evaluation of the severity of myocardial injury associated with mineralization. Future cases would benefit from concurrent biomarker assessment to better correlate the degree of tissue calcification with the extent of active myocardial damage.

## 4. Conclusions

In conclusion, this case presents a clinical and multimodal imaging assessment suggestive of metastatic myocardial and systemic calcification secondary to end-stage chronic kidney disease (CKD) in a feline patient. The synergistic interaction between severe metastatic calcification and uremic cardiomyopathy (UC) resulted in refractory restrictive diastolic dysfunction, leading to fatal congestive heart failure. A multimodal imaging approach was essential for an accurate diagnosis; while echocardiography was indispensable for evaluating hemodynamic consequences, computed tomography (CT) provided comprehensive visualization of the extensive mineral distribution, resembling a “virtual necropsy” across the myocardium, aorta, and extracardiac tissues.

The markedly elevated calcium–phosphorus product (CPP) in this patient underscores the critical need for proactive mineral management in cats with advanced CKD. Furthermore, the rapid clinical deterioration despite intensive therapy highlights the inherently irreversible and severe nature of established metastatic calcification. To the best of our knowledge, this report represents only the second detailed characterization of its kind in the feline literature, serving as a significant clinical reference for the diagnosis and prognostic assessment of cardiac calcification in cats.

## Figures and Tables

**Table 1:** Summary of serum biochemical and hormonal profiles at presentation.

Parameter		Value	Reference Range	Units
Renal & Electrolytes	Blood Urea Nitrogen (BUN)	140	17.6–32.8	mg/dL
	Creatinine	7.04	0.8–1.8	mg/dL
	Phosphorus	15	2.6–6.0	mg/dL
	Total Calcium	9.0	8.0–11.0	mg/dL
	Ionized Calcium (iCa)	1.05	1.11–1.38	mmol/L
	Potassium	5.61	3.3–4.5	mmol/L
Acid-Base & Proteins	Blood pH	7.16	7.21–7.41	-
	Total Protein	2.2	5.7–7.8	g/dL
	Albumin	0.8	2.1–3.3	g/dL
Hormonal Profile	Parathyroid Hormone (PTH)	123.7	2–14	pg/mL
	25-hydroxyvitamin D	49.5	100–150	ng/mL
Calculated Indices	Total Ca × P Product (CPP)	135		mg^2^/dL^2^
	iCa × P Product (iCPP)	63		mg^2^/dL^2^

## Data Availability

The data presented in this study are available in the article.
